# Developing an Electronic Frailty Index (eFI) and a biological age trajectory with a cohort of over one million older adults in Hong Kong

**DOI:** 10.1016/j.tjfa.2025.100021

**Published:** 2025-03-07

**Authors:** Tung Wai Auyeung, Carolyn Poey Lyn Kng, Tak Yeung Chan, Elsie Hui, Chi Shing Leung, James Ka Hay Luk, Kwok Yiu Sha, Teresa Kim Kum Yu

**Affiliations:** a Department of Medicine and Geriatrics, Pok Oi Hospital, Hong Kong; b Department of Medicine and Geriatrics, Ruttonjee Hospital and Tang Shiu Kin Hospital, Hong Kong; c Department of Medicine and Geriatrics, Kwong Wah Hospital, Hong Kong; d West Suffolk NHS Foundation Trust, United Kingdom; e Department of Medicine and Geriatrics, Caritas Medical Centre, Hong Kong; f Department of Medicine, Fung Yiu King Hospital, Hong Kong; g Department of Medicine and Geriatrics, United Christian Hospital, Hong Kong; h Department of Rehabilitation, Kowloon Hospital, Hong Kong

**Keywords:** Frailty, Frailty index, Electronic frailty index

## Abstract

**Background:**

Electronic health record (EHR) has been in place in many parts of the world. This fits in very well to the frailty index calculation proposed by Rockwood and thus a frailty index can potentially be generated automatically from an EHR database. Therefore, the Hong Kong Hospital Authority (HA) attempted to develop an electronic frailty index (HK eFI), by employing thirty-eight health variables from her own EHR database.

**Methods:**

Five cohorts of patients aged 60 years or above ever attended any services provided by the Hong Kong HA in the year 2015, 2016, 2017, 2018 and 2019, were included. The HK eFI trajectory with ageing, generated by the five cohorts, were compared to the one described by Rockwood's group. Following the UK eFI method, 4 levels of frailty were categorized, and they were examined whether they were related to mortality, readmission rate and hospitalization patient days.

**Results:**

Each successive cohort consisted of increasing number of patients aged 60 years or above. (2015, 1.14 million; 2016, 1.19 million; 2017,1.25 million; 2018, 1.31 million; 2019, 1.38 million). The gradients of the five trajectories ranged from 0.035 to 0.037, representing an increase in FI approximately 3.6 % annually. The intercept of the curves converged at 0.1, representing the FI at age 60 years was 0.1. Compared to the fit group, the adjusted hazard ratios of mortality of the mild, moderate and severe frail group were 1.77, 3.31 and 6.65 respectively and they were all statistically higher than the fit group. (*p* < 0.005) Likewise, there was a stepwise increase in readmission rate and hospital patient days utilization with increasing frailty levels.

**Conclusion:**

It is feasible to develop an eFI and a biological age trajectory from a large EHR database with local adaptation.

Frailty is a clinical condition characterised by the loss of biological reserves across multiple organ systems with ageing, rendering an individual vulnerability to decompensation after being challenged by a stressor [1]. To materialize this biological concept, Rockwood et al. [[2], [3], [4]] and Fried et al. [5] attempted to measure frailty, but with separate approaches. Rockwood et al. adopted a multiple deficit approach across multiple organ systems by employing the frailty index (FI) which is equal to the number of deficits divided by the number of health-related variables being surveyed [[2], [3], [4]]. In addition, they proposed this accumulation of deficits (or FI) as a proxy measure of ageing, that is a biological age surrogate [2]. On the other hand, Fried et al. employed an operational definition of frailty and quantify it by five dimensions, including unintentional weight loss, muscle weakness, slowness, exhaustion and inactivity [5].

Electronic health record (EHR) has been in place in many parts of the world to increase the efficiency and safety in health care delivery. The health data captured in the system usually covers a wide range of variables from clinical diagnoses, laboratory results, functional capacity, disabilities to social vulnerability. This fits in very well to the frailty index calculation proposed by Rockwood and thus a frailty index can potentially be generated automatically from an EHR database. It becomes a convenient method in measuring the frailty level of an individual. With that, Clegg et al. has developed and validated the first electronic frailty index in the United Kingdom (UK eFI) by using a large sample from a primary care EHR database [6]. Subsequently eFIs have been developed and validated in various countries and ethnicities [[7], [8], [9], [10], [11], [12]], different clinical settings [[7], [8], [9], [10],^12^] and disease groups [[13], [14], [15]]. They have also been demonstrated to be a valid predictor of adverse health outcomes [[13], [14], [15]].

The Hong Kong Hospital Authority (HA) is the sole public-funded health service provider in Hong Kong serving a population of over 7.6 million. This single service provider has a universal EHR database covering 42 hospitals and 112 primary and secondary care clinics all over Hong Kong. Her huge service volume provides a sufficiently large EHR database for the development of an electronic frailty index (eFI).

This report aims to describe how the Hong Kong Hospital Authority Frailty Index (HK eFI) and the biological age trajectory were developed using her own EHR database.

## 1 Methods

In March 2021, eight geriatrician leaders working in HA from the seven hospital clusters, covering the whole territory, were convened to lead the Work Group on the development of Electronic Frailty Index in Hong Kong. The Work Group consists of experts in geriatrics, nursing, allied health, statistics and data science, as well as primary and community services. Following the standard procedure in creating an eFI [16], these geriatrician leaders decided on the selection of clinically relevant health variables, representing frailty in older adults across multiple organ systems. The potential variables considered include functional measurements, diagnoses, laboratory results, and social status. The selection process also involved balancing the data relevance to frailty and the extent they were missing in the database. The prevalence of the variables and the prevalence trend with ageing was tested according to the rules in creating an eFI [7]. In the beginning, the Work Group identified 48 variables from our EHR database which were judged to indicate frailty. However, ten were subsequently excluded either because of low prevalence (less than 1 %) or because the prevalence did not rise with age with a non-positive regression coefficient. Fear of falls, timed up-and-go test, elderly mobility scale were excluded because their prevalence was low. Hepatitis B infection and depression were not selected because their prevalence did not increase with age. Eleven types of cancers, namely breast, uterine cervix, colorectal, corpus uteri, lung, liver, lymphoma, prostate, nasopharynx, ovary and stomach, were considered initially. However, the prevalence was too low and we decided to group them together as one item: any type of cancer. Therefore, in the end, only thirty-eight health variables remained selected. (See Appendix) During the whole process, this Work Group was supported by the Strategy and Planning Division of Hong Kong Hospital Authority Head Office for the advice on the statistical methods, the retrieval and the analysis of health variables for the creation of an eFI in the organization.

This project included five cohorts of patients aged 60 years or above ever attended any services provided by the Hong Kong HA in the year 2015, 2016, 2017, 2018 and 2019. The Work Group decided to compare the HK eFI trajectory with ageing, generated by the five cohorts, to the one described by Rockwood's group [2], in terms of the gradient and the intercept. In addition, by using the 2019 cohort, the quartile of the HK eFI was calculated by the UK eFI method, by dividing the 99 percentile of HK eFI by four [6]. Then the four quartiles of HK eFI were compared to the corresponding values of the UK eFI. Employing these 4 levels of frailty, the HK eFI was also examined whether it was related to mortality, readmission rate and hospitalization patient days. By using the 2016 cohort, survival analysis was undertaken to compare the four levels of frailty in terms of mortality and was surveyed from 1 January 2017 till 19 May 2021. The assumption of the Cox proportional hazard models was validated and it was then employed to calculate the hazard ratios of mortality without and then with adjustment for age and genders, using the fit group (eFI ≤ 0.125) as the reference group. The test was two-sided and any p-values less than 0.05 was taken as statistically significant. The 2019 cohort was separately employed to examine the unplanned readmission rate, defined as emergency admission within 28 days after discharge, and the hospital days utilized, surveyed for one year, from 1 January 2020 to 31 December 2020, among the four frailty levels.

This project has been approved by the Hong Kong Hospital Authority Research and Ethics Committee.

## 2 Results

**Fig. 1:**
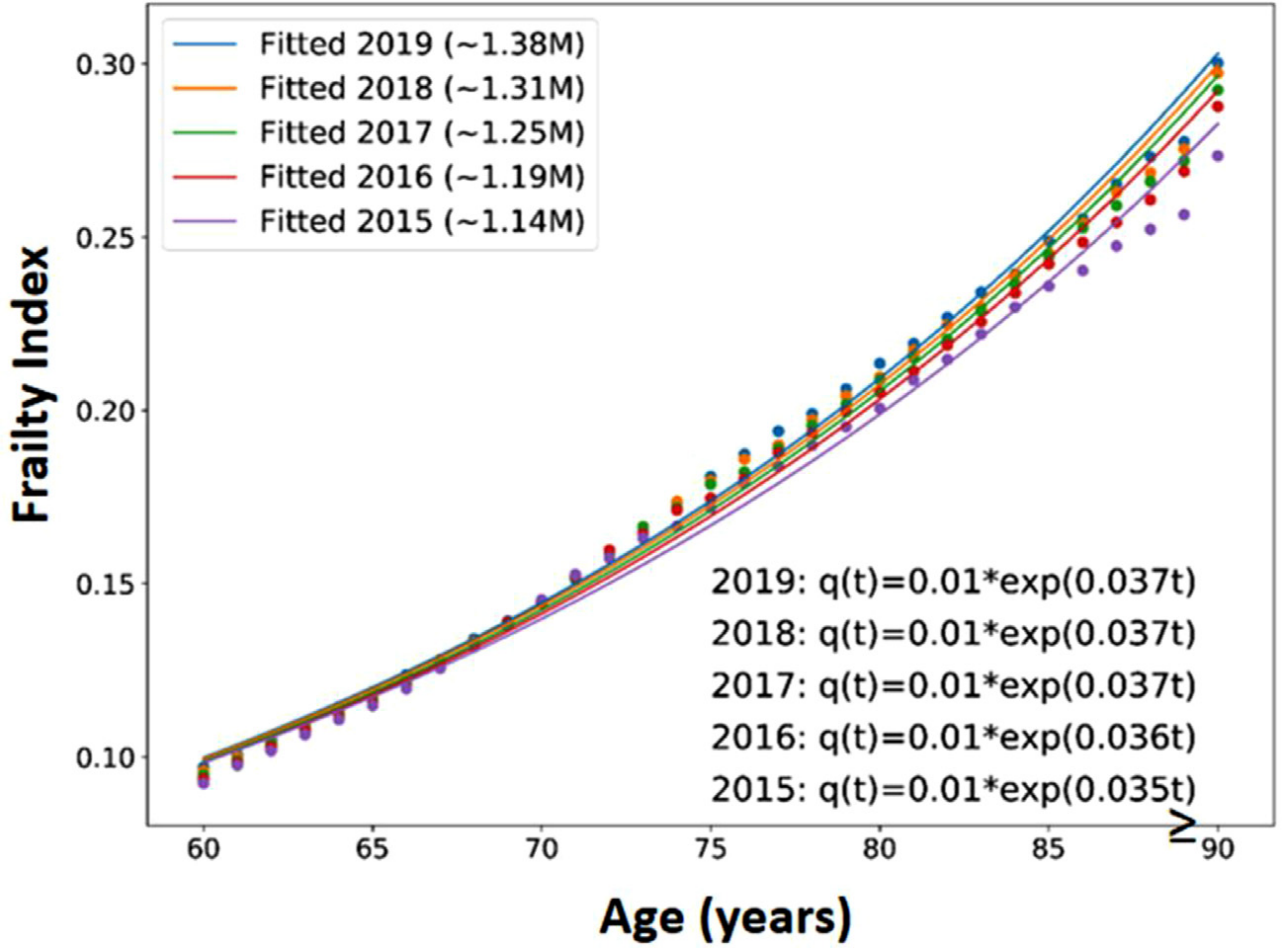
Frailty Index (Biological Age) trajectories of the five patient cohorts in HK eFI *t* = age(years).

With population ageing, each successive cohort consisted of increasing number of patients aged 60 years or above. (2015, 1.14 million; 2016, 1.19 million; 2017,1.25 million; 2018, 1.31 million; 2019, 1.38 million). For the latest 2019 cohort, 47 % were men and the mean age was 71.8 years (standard deviation, 9.2 years). The overall mean eFI was 0.16 (standard deviation,0.12); and it was 0.17 and 0.16 in men and women respectively. The deficit accumulation, or FI, was plotted against age (Fig. 1). The gradients of the five trajectories ranged from 0.035 to 0.037, representing an increase in FI approximately 3.6 % annually. The intercept of the curves converged at 0.1, representing the FI at age 60 years was 0.1.

**Fig. 2:**
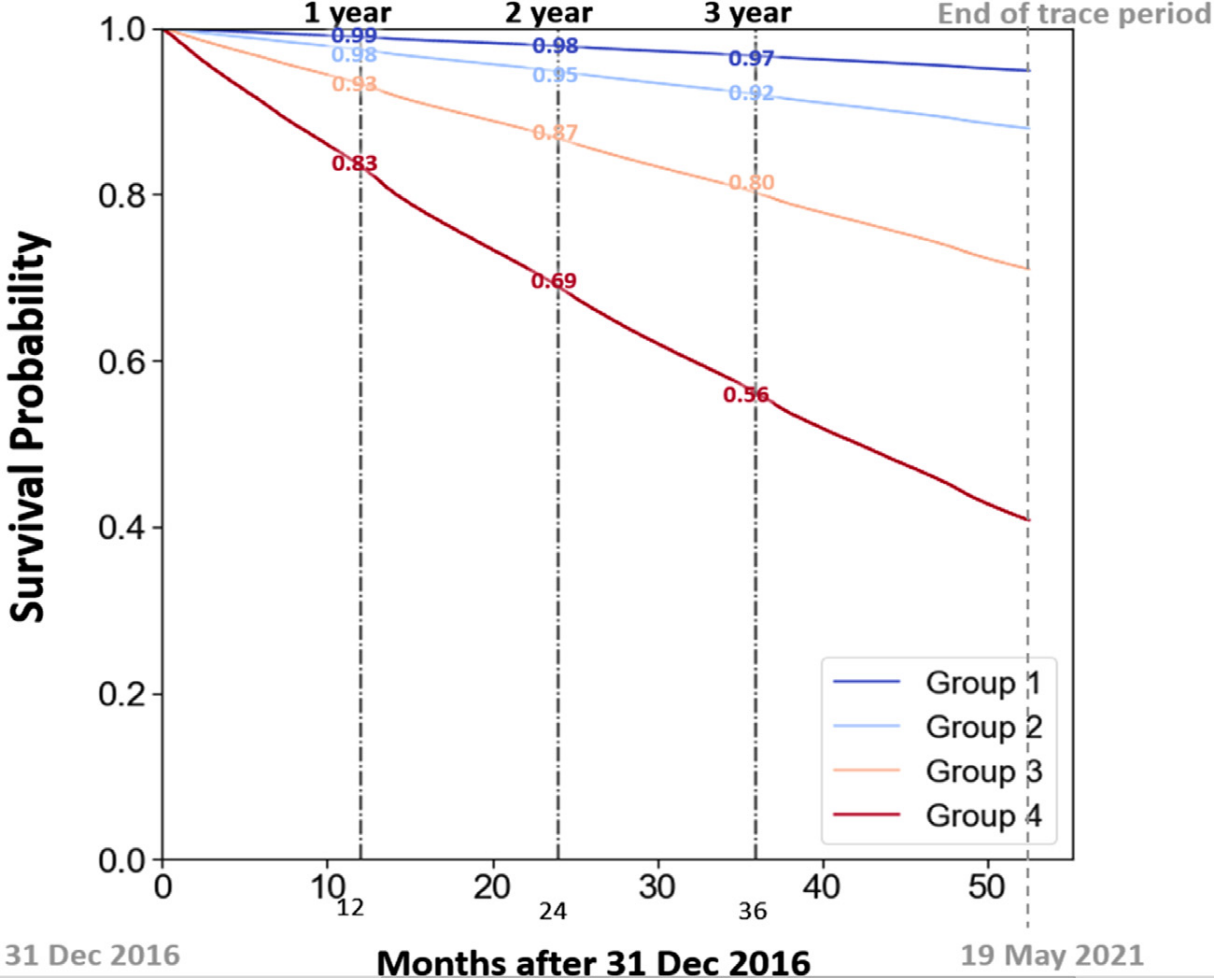
Survival Curves of the 2016 patient cohort categorized by four levels of frailty Group 1 (fit) FI ≤ 0.125; Group 2 (mild) FI > 0.125–0.25; Group 3 (moderate) FI > 0.25–0.375; Group 4 (severe) FI >0.375.

Using the 2019 cohort (N = 1.38 million), the 99 percentile was derived and was 0.5. Following the method of the UK eFI, the 99 percentile was divided by four to categorize the sample into four quartiles. Then each quartile was arbitrarily assigned as fit, mildly frail, moderately frail and severely frail [6]. The comparison between the quartile values and the three-year mortality rate in the UK eFI and the HK eFI were tabulated in Table 1. The quartile cut-off values of the HK eFI were close to but slightly higher than those of the UK eFI. The mortality rate of each HK eFI group was consistently lower than the UK eFI in each level of frailty level (Table 1). The relationship between the four frailty levels and mortality was examined by the Cox regression method in the 2016 cohort (N = 1.19 million). The four survival curves separate distinctly with the frailer groups demonstrating a steeper gradient (Fig. 2). Compared to the fit group, the adjusted hazard ratios (HR) of mortality of the mildly, moderately and severely frail group were 1.77, 3.31 and 6.65 respectively and they were all statistically higher than the fit group. (*p* < 0.005). The crude and adjusted HRs and 95 % confidence intervals were tabulated (Table 2). Likewise, we have tested the association between frailty level and readmission rate and hospital patient days utilization by using the 2019 cohort (N = 1.38 million). The results were listed in Table 3. There was a stepwise increase in readmission rate and hospital patient days utilization with increasing frailty levels (Table 3).

**Table 1:** Comparison between HK eFI and UK eFI.

	**HK eFI**	**UK eFI**
**99 percentile**	0.50	0.49
**Quartile interval**	0.125	0.120
**Fit**	0 – 0.125	0– 0.12
**Mild frailty**	>0.125 – 0.25	>0.12 – 0.24
**Moderate frailty**	>0.25 – 0.375	>0.24 – 0.36
**Severe frailty**	>0.375	>0.36
**Three-year Mortality**		
**Fit**	3.0 %	5.7 %
**Mild frailty**	8.0 %	14.0 %
**Moderate frailty**	20.0 %	28.6 %
**Severe frailty**	44.0 %	47.5 %

FI = frailty index.

**Table 2:** Three-year mortality comparisons by Cox Regression.

	Un-adjusted HR (95 % CI)	Adjusted HR (95 % CI
Fit	1.00	1.00
Mild frailty	2.45 (2.41–2.48)*	1.77 (1.75–1.79)*
Moderate frailty	6.51 (6.42 – 6.62)*	3.31 (3.26–3.36)*
Severe frailty	17.06 (16.79 – 17.33)*	6.65 (6.54–6.77)*

HR = Hazard Ratio; FI = frailty index; Fit, FI ≤ 0.125; Mild frailty, FI > 0.125–0.25; Moderate frailty, FI > 0.25–0.375; Severe frailty, FI >0.375; *p < 0.005; Adjusted for age and gender.

**Table 3:** Relationship between Frailty Level and Adverse Health Outcomes.

	**Unplanned readmission** **(number per 1000 person years)**	**Patient days in one year** **(days)**	**One year mortality (%)**	**Two-year mortality** **(%)**	**Three-year mortality** **(%)**
**Fit** **FI ≤ 0.125**	8.1	0.9	1	2	3
**Mild** **FI > 0.125–0.25**	25.5	2.1	2	5	8
**Moderate** **FI > 0.25–0.375**	80.2	5.1	7	13	20
**Severe** **FI >0.375**	216.4	10.6	16	31	44

FI = Frailty Index.

## 3 Discussion

Taking advantage of this sizable EHR database in Hong Kong, we have demonstrated that it is feasible to develop an automated eFI, and with it, to generate a biological age trajectory (Fig. 1). The gradient of the HK eFI biological age trajectory was 0.036, representing an average increase in FI approximately 3.6 % annually, which was close to the 3.5 % reported by Rockwood's group. [2]. The intercept of the curves converged at 0.1, representing the FI at age 60 years was 0.1. Again, this figure was similarly reported by Rockwood's group [2]. Therefore the biological age trajectory (Fig. 1) we generated was very close to the one developed by Mitnitski et al. [2].

The HK eFI not only is close to the Rockwood FI in terms of the deficit accumulation gradient and intercept (Fig. 1) but also approximates the UK eFI in terms of the quartile values (Table 1). This supports the notion that FI is independent of the health variables being selected provided that the sample is sufficiently large, and an adequate number of variables are selected across different organ systems and health dimensions [16]. Our sample are mostly ethnic Chinese and perhaps FI is also ethnicity independent as illustrated in the past studies [[7], [8], [9], [10], [11], [12]].

The quartiles cut-off values of the HK eFI, though close to, are slightly higher than those of UK eFI. It is plausible that our cohort covers from primary care to specialist ambulatory care and hospitalized patients. Despite being more frail, our cohort had a lower three-year mortality than the UK cohort. We have excluded deaths in the same year 2016 in this 2016 cohort and only surveyed death from 1 January 2017 onwards. It is uncertain whether this methodology variation can account for the observed difference. In addition, the health care system, life expectancy and the ethnicity difference may all contribute to the variation in quartile values of eFI and mortality.

The primary objective of this project is to test the feasibility of developing an eFI in Hong Kong and it was not research oriented. Therefore, the major pitfall is that the findings reported are only summary statistics generated from the Work Group meeting records. The Strategy and Planning Division did the analyses at the request of the Work Group and the results were presented to the Work Group during each meeting. The analyses were undertaken dynamically within the EHR system, and no permanent database was stored separately. This makes retrospective statistical analysis of our findings impossible after the Work Group had been resolved. The absence of a permanent data base, the lack of an external validation together with the retrospective analysis design can limit significantly the impact of our findings in this space of research.

Currently, to arrive at decision on invasive treatment, patients and clinicians rely on the average figure derived generally without considering the frailty status. With different FI, individual patient will carry different disease prognosis, and different risk-benefit ratios. Once the eFI of an individual patient is known, his or her biological age can easily be derived from the biological age trajectory (Fig. 1) we developed from our own population. Instead of interpreting the meaning of an eFI, this biological age information may appear easier to understand and apply when clinicians are considering the risk-benefit ratio of any aggressive intervention.

In future, this HK eFI still needs to be examined stringently whether it is capable to differentiate clinical outcomes in patients with different eFI. Only after this process, there will be sufficient evidence to support its use in informing the clinicians and patients for individualized management decision. Therefore, much research work is awaited to test the predictive accuracy of this eFI in various clinical conditions.

## 4 Conclusion

It is feasible to develop an eFI and a biological age trajectory from a large EHR database with local adaptation. Much work is awaited to provide sufficient evidence before it can be applied in various clinical conditions to inform the clinicians and patients the risk-benefit ratio in the consideration of aggressive therapy.

Declaration of Generative AI and AI-assisted technologies in the writing process.

Nil AI technology was used

## CRediT authorship contribution statement

**Tung Wai Auyeung:** Writing – original draft, Supervision, Project administration, Methodology, Formal analysis, Conceptualization. **Carolyn Poey Lyn Kng:** Writing – review & editing, Methodology, Formal analysis, Data curation, Conceptualization. **Tak Yeung Chan:** Writing – review & editing, Methodology, Investigation, Data curation. **Elsie Hui:** Writing – review & editing, Methodology, Investigation, Data curation. **Chi Shing Leung:** Writing – review & editing, Methodology, Investigation, Data curation. **James Ka Hay Luk:** Writing – review & editing, Methodology, Investigation, Data curation. **Kwok Yiu Sha:** Writing – review & editing, Methodology, Investigation, Data curation. **Teresa Kim Kum Yu:** Writing – review & editing, Methodology, Investigation, Data curation.

## Declaration of competing interest

On behalf of all authors, the corresponding author states that there is no conflict of interest.

## Appendix. Thirty-eight Variables of HK eFI

**Disease Diagnoses**

1. Coronary Heart Disease
2. Glaucoma
3. Hip Fracture
4. Hepatitis B Infection
5. Dementia
6. Hyperlipidaemia
7. Depression
8. Chronic Obstructive Pulmonary Disease
9. Atrial Fibrillation
10. Stroke
11. Chronic Kidney Disease
12. Diabetes Mellitus
13. Heart Failure
14. Hypertension
15. Parkinsonism
16. Any Cancer

**Sign and Symptom**

1. Fall Risk
2. Fall History
3. Fear of Falls
4. Memory / Cognitive Problem
5. Urinary Incontinence
6. Urinary Catheter
7. Bowel Incontinence
8. Underweight
9. Weight Loss
10. Polypharmacy (regular drugs > or = 5)

**Laboratory Tests**

1. Haemoglobin
2. Albumin
3. Creatinine

**Functional Impairment and Social Vulnerability**

1. Activity Limitation
2. Living Alone
3. Hearing Impairment
4. Mobility: Elderly Mobility Scale
5. Mobility: Timed Up Go Test
6. Mobility: Functional Mobility Ambulation Classification
7. Mobility Nursing Assessment
8. Requirement for Care
9. Visual Impairment
